# Expert Opinions on Best Practices for Overactive Bladder Management with onabotulinumtoxinA

**DOI:** 10.3390/toxins17040207

**Published:** 2025-04-21

**Authors:** Karyn S. Eilber, Benjamin M. Brucker, Andrea Pezzella, Vincent Lucente, Kevin Benson, Michael J. Kennelly

**Affiliations:** 1 Department of Urology, Cedars-Sinai Medical Center, Los Angeles, CA 90211, USA; 2 Departments of Urology and Obstetrics and Gynecology, New York University Langone Health, New York, NY 10016, USA; 3 Southern Urogynecology Wellness and Aesthetics, West Columbia, SC 29169, USA; 4 Axia Women’s Health, The Institute for Female Pelvic Medicine & Reconstructive Surgery, FPM Urogynecology Center, Allentown, PA 18103, USA; 5 Division of Gynecology, Section of Urogynecology, St. Luke’s University Health Network, Bethlehem, PA 18015, USA; 6 Department of Obstetrics and Gynecology, Lewis Katz School of Medicine at Temple University, Philadelphia, PA 19140, USA; 7 Department of Obstetrics and Gynecology, Division of Urogynecology and Pelvic Surgery, Sanford University of South Dakota Medical Center and Hospital, Sioux Falls, SD 57105, USA; 8 Department of Urology, Atrium Health Wake Forest University School of Medicine, Carolinas Medical Center, Charlotte, NC 28203, USA

**Keywords:** overactive bladder, onabotulinumtoxinA, urge incontinence, botox, procedure considerations, minimally invasive therapy

## Abstract

OnabotulinumtoxinA is an FDA-approved treatment for adults with overactive bladder (OAB) who have an inadequate response to, or are intolerant of, oral pharmacotherapies including anticholinergics or beta-3 agonists. However, procedural practices of onabotulinumtoxinA intradetrusor injection vary among practitioners and can affect patient experience. To address this, a panel of six high-volume intravesical onabotulinumtoxinA providers with 100 years of combined experience convened to discuss the best office practices when treating patients with OAB. These key best practices include counseling patients on available OAB therapies, including onabotulinumtoxinA, at the initial consultation in accordance with established AUA and SUFU guidelines in a way that is easily understood. An office setting is preferred over a hospital or surgery center when performing the procedure. Staff involvement, from scheduling to post-procedure, is essential for establishing the relationships necessary to optimize patient experience and encourage compliance and retreatment. Experts generally recommend using a viscous lidocaine bladder instillation for an anesthetic 15 min prior to the reconstitution of onabotulinumtoxinA with 5 to 10 mL of normal saline. A range of one to 20 injection sites is acceptable, with a smaller number preferred. Starting in the lower bladder, experts recommend using a slower speed of injection to improve distribution and decrease patient discomfort. Subsequent treatments should be regularly scheduled at six-month intervals with the option of re-treating earlier if symptoms return, but no sooner than 12 weeks. For office intravesical onabotulinumtoxinA procedures, optimization of the patient experience by the physician and their staff, starting with the initial visit through the post-treatment follow-up, is key to long-term patient compliance.

## 1. Introduction

Overactive bladder (OAB) is defined by the International Continence Society as urinary urgency usually accompanied by frequency and nocturia, with or without urge urinary incontinence [1]. OAB is a chronic condition that impacts patients’ quality of life and requires long-term management. The available treatment options for the treatment of OAB include non-invasive (mainly behavioral) therapies, oral pharmacotherapy, minimally invasive intravesical therapy, tibial or sacral neuromodulation, and surgical intervention (bladder augmentation). The American Urological Association (AUA) and the Society of Urodynamics, Female Pelvic Medicine and Urogenital Reconstruction (SUFU) emphasize the importance of shared decision-making to select the best therapy or therapies based on the patient’s needs, desires, and tolerance of side effects over the concept of “step therapy” [2].

OnabotulinumtoxinA (onabotA) is an FDA-approved, minimally invasive option for the treatment of adult patients with OAB and symptoms of urge urinary incontinence, urgency, and frequency [3]. The AUA guidelines recommend onabotA in patients who have failed an oral pharmacotherapy or for those who are unable or unwilling to undertake behavioral or pharmacological treatments. Studies have demonstrated that onabotA is safe and effective over repeated treatments that are typically readministered between 4 and 7.6 months [4,5,6].

While the clinical trials performed for the FDA approval of onabotA provide a framework for the procedure, individual practices vary [7]. Similar rates of clinical efficacy and adverse events have been reported for different injection paradigms, without clear recommendations [8,9,10]. Multiple non-inferior bladder analgesic options are available for procedural pain control [11], and there are few evidence-based recommendations regarding peri-procedural antibiotic prophylaxis surrounding onabotA treatment to reduce the risk of procedure-related urinary tract infection (UTI) [12]. While many clinicians embrace practice variability, this same variability may deter many practitioners from offering onabotA to their OAB patients. As a result, an expert panel including urologists and urogynecologists with high office volumes (10 to 20 intravesical onabotA patients per week) and with 100 years of combined experience convened to discuss their personal experiences and current research through moderator-led questions on focused topics in an effort to provide additional guidance for clinical practice. The experts were selected based on their credentials, years of experience, involvement in research, and reputations as experts amongst their peers. This paper summarizes the discussion from this group of six experts who routinely use onabotA to treat adults with OAB and describes best practices based on their opinions. The experts reviewed when and how to introduce onabotA to patients, effective approaches for the procedure day, and post-procedural care to allow for a positive treatment experience and improve long-term compliance.

## 2. Pre-Procedural Considerations

### 2.1. Introducing the Care Pathway

By the time a patient visits a specialist, they have typically tried one or more oral agent(s) that have been prescribed by another provider. After assessing and confirming the diagnosis of OAB, the health care professional (a physician or advanced care practitioner such as a physician assistant or nurse practitioner) should spend time introducing the OAB care options. The non-invasive (behavioral modification), pharmacologic, and minimally invasive therapies as recommended by the AUA/SUFU OAB guidelines should be discussed [2]. Patients and clinicians should talk openly about medication side effects, the potential risks of the various therapies, and issues such as accessibility and cost for patients in the US. It is important to start the conversation on the available therapies, including minimally invasive options such as onabotA, at the initial visit so that the patient is aware of the many therapeutic options for OAB to provide hope for finding a successful treatment [13]. Introducing onabotA early allows for patients’ familiarity with this treatment option and prepares them to more readily discuss it—if and when the time comes. It should be reinforced that regardless of the treatment a patient chooses, behavioral modification and fluid restriction are always important.

When initially explaining minimally invasive therapies, provide patients with key points and avoid making the discussion too complicated. The goal is to introduce the treatment options while keeping the explanations simple in order to give patients a sense of hope and a reason to return. For example, explain that there are effective options beyond diet and behavioral changes that, in some cases, may work more effectively than oral medications. Of the available minimally invasive therapies and taking into consideration patients’ preferences along with the therapy’s efficacy, ease of administration, mechanism of action, concomitant conditions, and cost, the experts view onabotA as a simple way to provide effective treatment for their patients. OnabotA is also a popular option with patients: when patients were asked to rank their preferences of minimally invasive therapies after being provided with the risks and benefits of each option, most chose onabotA as their first choice based on its long-term efficacy [14].

When introducing onabotA, highlight the therapy’s efficacy to give patients hope and expectations of success. In two Phase 3 clinical trials, onabotA significantly decreased the daily frequency of urinary incontinence episodes and improved all other overactive bladder symptoms compared to placebo [4,15]. Up to 31% of patients became completely continent, and the patient health-related quality of life was significantly improved [4,6,15]. Empower the patient not to settle—OAB is common, but it is not merely a normal part of aging [2]. Discussing the patient’s quality of life is important, especially for those who have a difficult time making treatment decisions. Focus on lifestyle improvements: Are they living their best possible life? There is always an opportunity for improvement. Experts typically set a realistic expectation of relieving patients’ symptoms by at least 75%, with the goal of becoming symptom-free. For patients in the US, explain that onabotA is typically covered by most insurance plans and that additional assistance can be provided for commercial insurance patients.

Move on to how it is administered, while using non-technical phrases that are easily understood, such as “it is placed in your bladder” [3]. When counseling on the risk of post-procedure incomplete bladder emptying, make sure to use patient-friendly language, such as “Nearly all patients could still urinate when they needed to, but some patients may not be able to fully empty their bladder” [3,16]. In the rare case of a patient needing to catheterize, explain that they do not need to have a “bag,” and describe self-management using clean intermittent catheterization (CIC) as an alternative way to drain the bladder with a small tube about the size of a coffee stirrer. Educate the patient that retreatment is usually every six months to prevent symptoms from returning, but it can be flexible depending on their schedule [3,4,5]. Most importantly, assure them that the provider and the staff at the practice will be with them through the entire treatment process to provide counsel at every step of the way. It is a partnership and not a journey for the patient alone. If the patient is not satisfied with onabotA or if they decide to move on to something else, other minimally invasive treatments can be considered as an alternative [2].

### 2.2. Shared Decision-Making: Determining Whether OnabotA Is the Right Therapy for the Patient

The patient’s preferences and logistical considerations can be critical to the treatment choice [2]. Non-medical factors often play a role in making recommendations for the patient. The provider should pose questions to understand their concerns, which may rule out alternative minimally invasive options such as neuromodulation: What is their lifestyle like? Is it feasible to return weekly for 12 weeks and then monthly for percutaneous tibial nerve stimulation (PTNS)? Are they technologically savvy enough to interact with a neuromodulation device that is implanted? How do they feel about a procedure that can require sedation or anesthesia? Are they willing to undergo repeated office procedures? OnabotA is a good option for patients unwilling to have minimally invasive surgery or staged treatment phases, which often have lifestyle restrictions. Many patients also tend to like that onabotA is not permanent. OnabotA should be an obvious choice for patients who absolutely do not want surgery, are not surgical candidates, and/or cannot return regularly for the maintenance of a neuromodulation device.

OnabotA is contraindicated in patients who have an active urinary tract infection, a known allergy to botulinum toxin or its components, or a post-void residual (PVR) over 200 mL who are not routinely performing CIC [3]. There are certain patient populations that may require special considerations, such as those who may be at a higher risk for incomplete bladder emptying and who are not performing CIC at baseline, including patients with diabetes or neurogenic bladder, those over 70 years old, and male patients with benign prostatic hyperplasia [3,17,18,19]. Body dystocia, arthritis, or any other physical disability resulting in the patient’s inability to physically catheterize should also be taken into consideration [3,4]. A summary of the benefits, potential side effects, and contraindications is provided in Table 1.

Using a holistic approach (Box 1), the provider should carefully assess each individual situation, be able to clearly explain why the therapy is being recommended, and use shared decision-making to determine what is best for the patient [2]. Most patients have already tried and failed other OAB treatments (including multiple medications) and are looking to you for guidance.

Box 1 **A holistic approach to prescribing treatments for OAB.**

A holistic approach to prescribing treatments for OAB considers not only the medical condition but also the patient’s unique circumstances [2]. Consider the patient’s viewpoint when evaluating the options, given their unique circumstances, including psychosocial, economic, social support, and educational factors.Psychosocial Factors.Patient Preferences: Involve patients in decision-making when discussing treatment options.Cultural Beliefs: Cultural context may affect acceptance of and/or adherence to certain therapies.Social Support: Consider the patient’s support system and ability to manage medications and appointments.Economic Factors for US PatientsAffordability: Assess whether the patient can afford the prescribed medication.Insurance Coverage: Check whether the drug is covered by the patient’s insurance plan.Practical ConsiderationsRoute of Administration: Choose an appropriate form (oral, injectable, topical).Frequency: Consider dosing frequency and convenience for the patient.Storage: Ensure patients can store and handle the medication properly.Adherence and EducationPatient Education: Provide clear instructions, potential side effects, and warnings.Monitoring: Regularly assess treatment effectiveness and address any issues.The experts have felt that onabotA is their “go-to,” as it does very well with the holistic approach, taking all of these factors into account.

### 2.3. Where Should the Procedure Take Place?

OnabotA procedures can be performed in the office, an ambulatory surgery center, or a hospital surgery suite; however, the experts feel that for the optimal patient acceptability of long-term onabotA treatment, the procedures should be performed in the office [20]. Establishing a well-run, office-based onabotA practice ensures efficiency for both patients and providers. It considers the patient’s perspective regarding cost, time management, and adherence. As the actual time spent by the physician on the onabotA injection is typically only a few minutes, time can be lost by traveling to another location and/or by surgical delays. For the provider, best practices are easier to tailor in an office setting and makes post-procedural follow-up more practical.

For a minority of patients, there may be exceptions or mitigating circumstances that make a location other than the office preferable. Office procedures may be impractical for some patients due to their pain intolerance or inability to position themselves without significant pain or because of a physical disability. Using a facility that offers sedation may be more desirable for patients who have medically complicated situations or those with OAB caused by a neurological condition. Moreover, not all providers can perform the procedure in the office; for example, they may lack equipment such as cystoscopes. The use of an ambulatory surgery center or hospital operating room not only accommodates these situations but also offers providers who are new to injecting onabotA the opportunity to build this skill set. Performing the first few onabotA procedures with sedation is a great way to gain experience and help transition the provider to the office setting.

### 2.4. Logistics of Practice: Are You Set Up for Success?

The logistics of practice are essential for providers who are just starting to use ona-botA. Regarding access, is your office set up to accommodate a certain volume of patients in a timely manner? Can your office or procedure room handle repeat procedures? Regarding the equipment and setup, consider the number of cystoscopes available. Single-use, disposable cystoscopes can be an effective, safe, and affordable alternative to reusable scopes to accommodate a high volume of patients [21,22]. Single-use cystoscopes also reduce the need for the staff to process equipment or to delay or limit procedures based on availability. Regarding onabotA inventory, consider the financial and storage capacities of your practice. Assess the need for a buy-and-bill process and the ability to properly store onabotA, which also extends to inventory management and expiration date tracking.

## 3. Day of Procedure Considerations

### 3.1. Emphasizing the Patient Experience

The experts emphasize that the force driving the overall success of repeat treatments is the patient’s experience. The atmosphere should be comfortable and appealing from the moment the patient walks into the office to the conclusion of the procedure. There are several considerations that can help with patients’ comfort and anxiety, such as dimming the lights, letting the patient choose the music, and offering a blanket and/or aromatherapy. A recent study on the use of aromatherapy and music during magnetic resonance imaging procedures suggested these could reduce anxiety and improve patients’ comfort [23]. Anxiety can also be reduced by keeping items used for the procedure, such as the cystoscope, out of the patient’s view. Distracting the patient with ongoing dialogue is also useful [24]. Encourage patients to keep their eyes open during the procedure, as it requires greater central nervous system processing of vision, which can decrease pain perception [25]. It may be beneficial to encourage patients to take an over-the-counter oral analgesic or nonsteroidal anti-inflammatory medication upon leaving their residence for the procedure.

Be consistent in your procedural flow, as patients will become used to the process and have proper expectations. Having a favorable environment and a relaxed patient also benefits the medical assistants. Soliciting patients’ feedback is a great way to keep finding new ideas for process improvements and to incorporate patients’ preferences for repeat visits. Patients appreciate when providers make an obvious effort to ensure their comfort. They value the personal touches. Interpersonal interactions are important, and continuity with regard to staff is key. According to the experts, they estimate that approximately 70% of their patients come back for retreatment based on a positive experience with the staff, which may be challenging for practices that have high turnover or different staff members on irregular schedules. When consistency in the staff is not possible, additional staff training or referencing documented patient feedback for procedural preferences may be an alternative. Patients like to feel that the staff are their friends and that they can see a familiar face at each visit. Demonstrated care for patients’ wellbeing and comfort by the front desk staff and schedulers can help with the feeling that appointments are transactional. For example, having the same nurses perform the follow-up and the two-week post-procedure check-in not only allows the staff to assess how the patients are doing but also enables the patient to experience a true connection. These interpersonal relationships add to the sense of lifestyle improvement. When you get to know the patient, the resulting trust and care they feel play a major role in giving them a reason to return for all procedures in your office, not just for the onabotA treatments.

Regarding staff retention, when staff members feel connected, they are also more likely to stay. In a larger organization, a sense of ownership by the staff members can become lost if there is no clear direction on individual responsibility. Physicians have a true leadership responsibility in this area, taking the reins and outlining the expectations. These can be verbalized or written down. While care maps are frequently utilized for patients, they are also needed for the staff. How does your staff fit into the overall process? Make their roles clear. During staff training or when onboarding new staff members, emphasize the focus on the patient experience over efficiency. The staff truly shapes the experience, so encourage them to have a sense of ownership and make them feel empowered. Allow your staff to take control of setting up the procedure room, informing patients about antibiotics, and being there to hold their hands during the procedure. The physician may be the injector providing the treatment, but it is the staff that sustains the positive, service-based experience.

### 3.2. Obtaining Consent

In the US, state and local requirements may vary, but patient consent should be obtained before each procedure. After the original consent form is signed, some experts suggest obtaining an abbreviated consent by having patients re-initial and date the original consent on each subsequent visit. Others prefer to require renewed consent with each round of onabotA and a review of the risks and benefits, which allows patients to have their questions answered prior to the procedure. This step should be individualized to satisfy the requirements and balanced with efficiency to ensure that providers have sufficient time to maximize their productivity.

### 3.3. Pre-Procedure Urinalysis

The expert opinions vary on the use of pre-procedure urinalysis (UA), and it is ultimately left to the discretion of the clinician. Performing a UA prior to the procedure day when a patient has no acute urinary tract infection (UTI) complaints can lead to an unnecessary cancellation if there are UA findings but the patient is asymptomatic. However, for some clinicians, the presence of asymptomatic bacteriuria may deserve further discussion and/or intervention. Some experts recommend performing a dipstick test on the day of the procedure, but this could potentially lead to aborted procedures, so experts who recommend this also prescribe pre-procedure antibiotics (see next section). Some suggest following the AUA guidelines for asymptomatic bacteriuria screening [26]. Others simply check whether the patient is symptomatic by asking, “Do you have burning with urination?” Symptoms suggestive of a UTI would prompt a UA before the procedure. Some experts who do not perform a UA will delay reconstituting onabotA until they have visualized the bladder by cystoscopy and will only proceed if the bladder mucosa has a normal appearance, which can rule out a local inflammation suggestive of an active infection. The experts agree that the procedure must be cancelled if there is an active infection.

### 3.4. Prophylactic Periprocedural Antibiotics

The experts agree that some form of a prophylactic antibiotic may be beneficial to ensure the patient does not arrive on the procedure day with a UTI, resulting in an aborted procedure, or that a subclinical UTI does not progress after the procedure is performed. The choice of an antibiotic can be made based on the patient’s allergies and the AUA guideline for a cystourethroscopy with manipulation that recommends a single dose of either a fluoroquinolone, trimethoprim-sulfamethoxazole, aminoglycoside, first- or second-generation cephalosporin, or amoxicillin-clavulanate [26]. However, caution is warranted for the concomitant use of onabotA and aminoglycosides [3]. One expert recommends giving a 3-day course of antibiotics starting on the day before the procedure, while others administer an oral antibiotic right before or immediately after the procedure as one dose with success. This single dose is convenient for patients, saving them a trip to the pharmacy. For patients with a history of recurrent UTIs, or for those traveling a greater distance who could be impacted by cancellation/rescheduling, antibiotics could be considered at least one day before the procedure. Antibiotic stewardship should be considered, especially in older patients. Taken together, there is some variability in the timing of the prophylactic antibiotic course used by the experts based on the individual patient circumstances since the data varies on whether antibiotic use, regimen, and route impact the post-procedure UTI rates [27].

### 3.5. Discontinuation of Anticoagulants and Antiplatelets

Anticoagulants and antiplatelets may be discontinued prior to the onabotA procedure, but it is not required [28]. The experts agree that the use of smaller gauge needles and the proper direct visualization of the blood vessels allow for safe procedures when patients are on blood thinners. Ultimately, it is left to the discretion of the provider and the consideration of the bleeding risk.

### 3.6. Local Analgesia or Anesthesia for the OnabotulinumtoxinA Procedure

Oral phenazopyridine may be administered the night before and the morning of the procedure, as a substitute or in addition to topical intravesical lidocaine to enhance patient comfort [11]. Patients using phenazopyridine should be instructed to anticipate orange urine that may stain clothing. Experts recommend using a viscous lidocaine bladder instillation at least 15 min before the onabotA procedure. One expert recommends pre-packaging supplies ahead of time and having everything pre-mixed to allow for the lidocaine instillation as quickly as possible after the initial intake. During the instillation dwell time, vitals can be taken, the UA may be processed if a sample was taken, and a patient preference sheet can be completed to recreate or improve the experience for the next appointment. The experts proceed with the onabotA procedures with the lidocaine remaining in the bladder to improve the procedure’s overall efficiency and based on a lack of evidence to the contrary.

### 3.7. Type of Cystoscope

Both rigid and flexible cystoscopes are appropriate for use in onabotA procedures, with different advantages for each [4,29]. The flexible cystoscope is a two-person procedure, while the rigid cystoscope is a one-person procedure. With a rigid cystoscope, the medical assistant can be focused on ensuring patient comfort, such as by holding the patient’s hand while having an engaging conversation. If an assistant is needed for the injection process, seamless communication is required for the proper injection timing. If you have a new medical assistant or other personnel requiring verbal communication, set the expectation with the patient ahead of time so that they are comfortable with the verbal exchange.

### 3.8. Injection Paradigms: Dose, Location, Volume, and Number of Injection Sites

Over the years, many injection paradigms have been explored. The onabotA United States Prescribing Information (USPI) details a 20-injection site paradigm using a total dose of 100 Units (U) as 0.5 mL (5 U) injections across 20 sites into the detrusor [3]. The Phase 4 LO-BOT study evaluated an alternative injection paradigm using 100 U of onabotA reconstituted with 5 mL of normal saline and administered as 10 injections of 0.5 mL—two in the trigone and eight distributed evenly across the posterior wall of the bladder around the trigonal area [30]. The use of this paradigm resulted in improved treatment benefits over placebo with a low incidence of incomplete bladder emptying requiring CIC.

The optimal number of injections maximizes the distribution while minimizing the number of injection sites. The experts recommend a range from one to 20 injection sites based on experience and available data, balancing the number of injections with efficacy and patients’ comfort [3,10,30,31,32]. Most of the experts recommend fewer injections while balancing the fact that onabotA must be adequately taken up by the peripheral nerve terminals. The expert panel agrees that the number and location of injections can be customized for each patient. Based on a meta-analysis, trigonal-involved onabotA injections were more effective compared with trigone-sparing injections [33], likely due to the dense innervation of the trigone [34]. Ultimately, the decision should be made at the time of the injection based on the patient’s comfort and technical considerations such as the patient’s anatomy and the ease of reaching all of the intended injection sites.

When performing a minimal injection paradigm, the experts recommend initiating the first dose of onabotA at 100 U, with a reconstitution volume of 5 to 10 mL and using 0.5 to 5.0 mL per injection site, based on diffusion [3,35]. A dose titration up to 200 U may be considered in patients who have an inadequate response and minimal side effects [2]. When patients return, information on symptomatology can be correlated with the dose, volume, and number of injections, and adjustments can be made accordingly. This will allow for the reevaluation of optimal diffusion, with the objective of reaching more presynaptic nerve terminals.

### 3.9. Depth and Rate of Injections

The experts agree that the depth or plane of injection is important for the efficient lateral distribution of onabotA, especially when attempting to use fewer injection sites. The afferent nerves are located in the superficial suburothelium and are appropriate to target for OAB dry (without urge incontinence) [36], while the efferent nerves are in the deeper detrusor section of the urothelium and are appropriate for OAB wet (with urge incontinence) [37]. Adding a drop of dye such as sodium fluorescein is an off-label suggestion to optimize the technique, and it may help visualize the outline of the injection and enable a better determination of the correct injection depth that is not too superficial [38]. A “groundswell” should be visible with minimal pressure. Adjust the needle if necessary to maintain an optimal depth, as too superficial of an injection results in a bleb [29], which is not desirable. The rate of infiltration is also an important consideration; the slower the rate, the greater the dispersion, with less pain experienced. Force should never be used since it generates more pressure. A slower infiltration also allows for easier adjustments if the needle is at the wrong depth, as evidenced by a loss of the onabotA out of the injection site and/or the development of a bleb.

## 4. Post-Treatment Considerations

### 4.1. Management of Adverse Events

Incomplete bladder emptying leading to an elevated PVR has been observed following onabotA treatment, and in some cases, this may require temporary treatment using CIC [3,17]. Most of the experts recommend only performing a PVR test once, two weeks after the initial treatment. A post-procedure PVR after subsequent treatments is considered generally unnecessary unless the patient has had a dose increase, if there is a lack of improvement in OAB symptoms following the procedure, or if the patient had a significantly elevated PVR (generally with symptoms of incomplete emptying) after their first treatment. In consideration of the age-based risk stratification for the incidence and duration of post-procedural CIC [39], some of the experts tend to be more liberal with younger patients but more conservative with older patients when determining the need for a post-procedural PVR. The experts agree that patients with a PVR >250 mL do not necessarily need to perform CIC, but the ultimate decision can be made based on the patient’s symptoms and the overall clinical picture [16].

The onset of onabotA is approximately 1–2 weeks [40]; therefore, any OAB medications can be discontinued after one week of the procedure with a minimal risk of retention. However, patients should be instructed to discontinue them sooner if voiding difficulty ensues.

### 4.2. Retreatment

Make a conscious effort to set up follow-up appointments. The office staff can help navigate the patient back for additional treatments. Most experts agree that patients should be proactively scheduled for their next procedure before leaving the office rather than waiting until they have symptoms to avoid a significant delay for subsequent procedures and/or the return of symptoms. There is variability in the duration of patients’ responses; for some, symptoms may return in four months, and for others, they do not return for eight months to a year [3,4,5,6]. Regardless of when symptoms return, most of the experts schedule a follow-up treatment six months following the first procedure to avoid having time without effective therapy. Setting up retreatment visits every six months is common practice, and appointments can be rescheduled earlier if symptoms return sooner. Six months is consistent with the retreatment paradigms of the pivotal onabotA clinical trials and the open label extension studies [3,4,5,41]. Patients who are traveling or have an upcoming special event to attend may receive reinjections earlier, but no sooner than 12 weeks after their last procedure [3].

## 5. Final Thoughts

While onabotA was approved by the FDA in 2013 for the treatment of adults with OAB, procedural practices still vary among practitioners, and clinicians often seek advice on how to appropriately manage patients with OAB using onabotA. Table 2 summarizes the recommendations of an expert panel of providers with 100 years of combined intravesical onabotA experience in an effort to provide additional guidance and recommendations for clinical practice.

## Figures and Tables

**Table 1:** Benefits, potential side effects, and contraindications of onabotA for OAB.

Benefits
Most patients have at least 75% relief of symptoms Up to 31% of patients became completely continent in clinical trials Long-term safety and efficacy Significant improvements in QOL Reversible Infrequent (average twice yearly) office treatments
Potential Side Effects
UTI Temporary inability to void Dysuria Increased residual urine volume
Absolute Contraindications
PVR > 200 mL in those not routinely performing CIC Active UTI Hypersensitivity to botulinum toxins or any of the components in the formulation

CIC = clean intermittent catheterization; OAB = overactive bladder; PVR = post-void residual; QOL = quality of life; UTI = urinary tract infection.

**Table 2:** Summary of expert recommendations.

Category	Recommendations
Pre-procedural considerations	Discuss OAB care pathway using simple language Perform the onabotA procedure in an office, not a hospital Ensure practice is set up for success
Day of procedure considerations	Emphasize patient experience
	Check for UTI symptoms, perform UA if needed Use prophylactic antibiotics Use lidocaine gel 15 min before procedure Reconstitute 100 U vial with 5 to 10 mL 1 to 20 injection sites, balance comfort and diffusion Slow instillation reduces pain
Post-procedural considerations	Perform 2-week PVR once after initial treatment unless dose is increased Proactively schedule next procedure every 6 months Move up appointments if symptoms return sooner

OAB = overactive bladder; PVR = post-void residual; U = units; UA = urinalysis; UTI = urinary tract infection.

## Data Availability

No new data were created or analyzed in this study. Data sharing is not applicable to this article.
